# Exploration of Hub Genes in Retinopathy of Prematurity Based on Bioinformatics Analysis of the Oxygen-Induced Retinopathy Model

**DOI:** 10.1155/2022/9835524

**Published:** 2022-09-10

**Authors:** Qi Xiong, Zhiliang Li, Jing Zhang, Lin Yang, Xiaomin Chen, Yan Gong, Xiaojun Cai, Min Ke

**Affiliations:** ^1^Department of Ophthalmology, Zhongnan Hospital of Wuhan University, Wuhan 430071, Hubei, China; ^2^Department of Orthopedics, Renmin Hospital of Wuhan University, Wuhan 430024, Hubei, China; ^3^Department of Biological Repositories, Zhongnan Hospital of Wuhan University, Wuhan 430071, Hubei, China

## Abstract

Retinopathy of prematurity (ROP) is a major blindness-causing disease that is characterized by an arrest of normal vascular development and neovascularization of the retina. Previous studies have shown that genetic factors may be associated with the development and severity of ROP. However, the genes and mechanisms underlying ROP remain unclear. We aimed to identify hub genes in ROP and drugs related to these genes by integrative analysis. The expression profiles of GSE158799 and GSE135844 were acquired from the Gene Expression Omnibus (GEO) database, and differentially expressed genes (DEGs) were identified. Then, an integrative analysis was performed including Gene Ontology (GO), Kyoto Encyclopedia of Genes and Genomes (KEGG), gene set enrichment analysis (GSEA), protein-protein interaction (PPI) network, transcription factor (TF)-gene, and miRNA-gene networks analysis. Moreover, we verified hub genes and identified potential drugs. 225 common DEGs were identified. Biological function analysis indicated that angiogenesis, cell surface, cell adhesion, extracellular matrix, and focal adhesion genes were enriched among DEGs. The PI3K/Akt signalingpathway, focal adhesion, and extracellular matrix (ECM)-receptor interaction were markedly enriched in the KEGG pathway analysis. Finally, 5 hub genes related to the nosogenesis of ROP were identified and found to be targeted by VEGFA inhibitors, TLR4 antagonists, and sunitinib. The present study showed that VEGFA, ACTA2, MKI67, CD68, and TLR4 are potential hub genes involved in the pathogenesis of ROP. Moreover, TLR4 antagonists and sunitinib may be new candidate drugs for ROP therapy, in addition to VEGFA inhibitors.

## 1. Introduction

Retinopathy of prematurity (ROP) is an abnormal vascular proliferation of the retina that occurs in preterm infants, especially low birth weight infants, and is the primary cause of childhood blindness worldwide. In recent decades, with the increasing incidence of prematurity and advances in medical technologies capable of rescuing the complications of premature infants, the incidence of ROP has increased dramatically and is becoming a growing serious clinical and social problem in contemporary pediatric care [1]. Regular fundus examinations are routinely performed in premature infants to allow early detection and timely treatment of ROP to reduce future visual impairment. Nevertheless, some preterm infants develop serious ocular complications, such as retinal detachment. ROP is a multifactor disease, and its incidence is known to be influenced by gestational age, birth weight, and environmental factors such as oxygen supplementation. However, none of these acknowledged risk factors can explain why some infants develop ROP and others do not. Neither of these factors can explain why some infants who develop ROP progress to severe ROP, while most experience disease resolution. In recent years, genetic factors have been verified to be associated with the development and severity of ROP in multiple studies [2–4]. Encouragingly, a study of monozygotic and dizygotic twins indicated that approximately 70% of the risk for ROP can be attributed to genetic factors [5]. Oxygen-induced retinopathy (OIR) phenotypes in rodent models also revealed proof of genetic effects, with discrepancies in retinal avascular areas and VEGF expression found between different rat strains [6–8]. An increasing number of researchers have reported that multiple genes are involved in predisposition to ROP in animal models and humans, and numerous candidate gene-related studies have been performed [9–13]. However, which genes are crucially and closely correlated with ROP is still unclear. Therefore, it is urgent to explore the possible candidate genes and pathophysiological mechanisms of ROP to provide new strategies for the screening and treatment of ROP.

With the rapid development of genetic technology, gene chips and sequencing have been extensively applied in many disease contexts, and the corresponding data are stored in public databases. Researchers can analyze the gene expression profile data to identify potential candidate biomarkers. Gene expression profiles can be downloaded from the public Gene Expression Omnibus (GEO) database for high-throughput experimental data. This approach allows us to better elucidate the pathogenesis of ROP by enabling comprehensive analysis at the multiple gene level. Integration and reanalysis of these genetic data provide the opportunity to identify biomarkers for ROP.

Recently, some studies have used microarray data profiles to illustrate the pathogenesis of ROP [9–14], but these studies were based on only a single cohort or single genetic event. To overcome this inadequacy, a comprehensive combination of gene expression profiling techniques and integrative bioinformatics approaches should be performed. In our research, we analyzed two microarray datasets (GSE135844 and GSE158799) from GEO, including gene expression data for mouse retina samples from 6 controls and 6 OIR models. The differentially expressed genes (DEGs) between the OIR and control samples common to both datasets were obtained. Then, to probe the molecular mechanisms of ROP, biological function and pathway enrichment analyses were performed. Gene set enrichment analysis (GSEA) was carried out to analyse the mechanism by which the target genes may affect the pathogenesis of ROP. Protein-protein interaction (PPI) network analysis was performed on the STRING website to identify potential hub genes. Additionally, a TF (transcription factor)-gene network, miRNA-gene network, and drug-gene network were constructed. Finally, to further validate the identified hub genes, RT-qPCR was conducted on RNA extracted from peripheral blood samples collected from premature infants.

## 2. Methods

### 2.1. GEO Datasets

**Figure 1:**
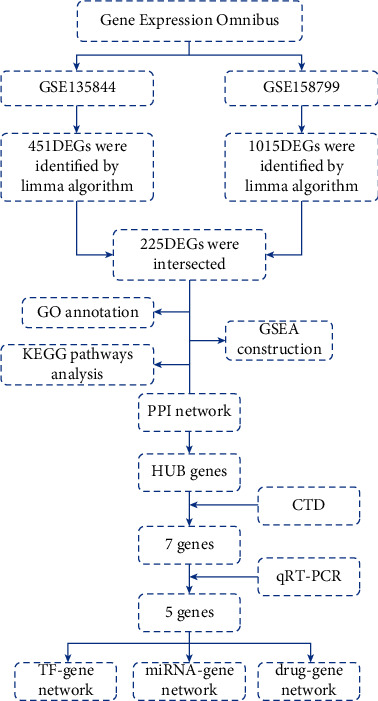
The flowchart of the research process. GO, Gene Ontology; GSEA, gene set enrichment analysis; KEGG, Kyoto Encyclopedia of Genes and Genomes; PPI, protein-protein interaction.

Two gene expression profiles (GSE158799 and GSE135844) were downloaded from the GEO dataset (https://www.ncbi.nlm.nih.gov/geo/). In both datasets, mice in the OIR group were subjected to 75% oxygen from postnatal day 7 to postnatal day 12 and then transitioned to room air conditions (21% oxygen), while the control group was kept in room air (21% oxygen) for the entire duration of the experiment. All mice were euthanized on postnatal day 17. The entire experimental process is illustrated in a flowchart (Figure 1).

### 2.2. Differentially Expressed Gene (DEG) Identification

The original data were standardized using the affy and LIMMA packages in *R* (version 4.0.5). The DEGs in OIR and control samples were identified, and the selection criteria were set as |log_2_ FC| ≥0.585 and adjusted *P* value < 0.05. All DEGs were displayed using volcano maps and heatmaps as intuitive visual presentations. Finally, the DEGs common to the two datasets were identified by a Venn diagram web tool (https://bioinformatics.psb.ugent.be/webtools/Venn/).

### 2.3. GO and KEGG Enrichment Analysis

Gene Ontology (GO) and Kyoto Encyclopedia of Genes and Genomes (KEGG) analyses were performed through DAVID (https://david.ncifcrf.gov/), which is a database for the functional interpretation of genes or proteins and can be used for the annotation and visualization of bioinformatics data [15–17]. *P* < 0.05 was considered statistically significant enrichment. The results are shown in a bubble chart made in *R*.

### 2.4. Gene Set Enrichment Analysis

GSEA is used to computationally analyze the difference in defined gene sets between two groups. Here, GSEA software (version 4.1.0) was used to analyze the differences in the gene sets of the OIR group and the control group [18]. We set the permutation number to 1000. A *p* value < 5% and a false discovery rate (FDR) < 25% were the criteria for a gene set to be considered enriched.

### 2.5. PPI Network Construction and Hub Gene Identification

We constructed the PPI network among 225 DEGs by using STRING (https://string-db.org). The confidence score >0.7 was considered the cutoff point. Cytoscape (version 3.8.0) was utilized to draw the diagram of the PPI network [19, 20]. The significant gene modules were identified through the MCODE plugin. The parameters were set as follows: MCODE score ≥5, node score cutoff = 0.2, k-score = 2, degree cutoff = 2, and max depth = 100. The hub genes were screened according to the node degree by the CytoHubba plugin. Then, the Comparative Toxicology Database (CTD), which catalogs disease–gene interactions, was used to identify the hub genes that were closely related to ROP [21].

### 2.6. Hub Gene Validation

The collection and examination of peripheral blood samples from premature infants in our work were performed with permission from the Ethics Committee of Zhongnan Hospital of Wuhan University. We collected peripheral blood from 10 premature infants (5 ROP and 5 non-ROP). Total RNA was isolated from each peripheral blood sample with TRIzol reagent (Invitrogen, USA). Then, the obtained RNA was transcribed into cDNA. RT-qPCR assays were performed using SYBR Green mix (Toyobo, Japan) in a StepOnePlus real-time qPCR system (Applied Biosystems, CA). All the acquired data were analyzed using the 2^−ΔΔCT^ method and normalized to the levels of housekeeping gene GAPDH. The primer sequences used for RT-qPCR are shown in Table 1.

### 2.7. TF-Gene and miRNA-Gene Network Construction

Under certain disease conditions, miRNAs or TFs regulate gene expression via post-transcriptional interactions with target genes [22]. TF-gene and miRNA-gene networks were constructed using the NetworkAnalyst database (https://www.networkanalyst.ca/) and visualized with Cytoscape software [23].

### 2.8. Candidate Drug Identification

Drugs or molecular compounds that may interact with the identified hub genes were detected through DGIdb (https://www.dgidb.org), an academic platform for predicting the interaction between drugs and sensitive genes [24]. Cytoscape software was used to visualize the drug-gene interaction network.

### 2.9. Statistical Analysis

Statistical analysis was performed using SPSS 22.0 (SPSS Inc., USA). Student's t-test was used to analyze the differences in gene expression in peripheral blood between the non-ROP group and the ROP group. *P* < 0.05 was considered statistically significant.

## 3. Results

### 3.1. DEG Identification

**Figure 2:**
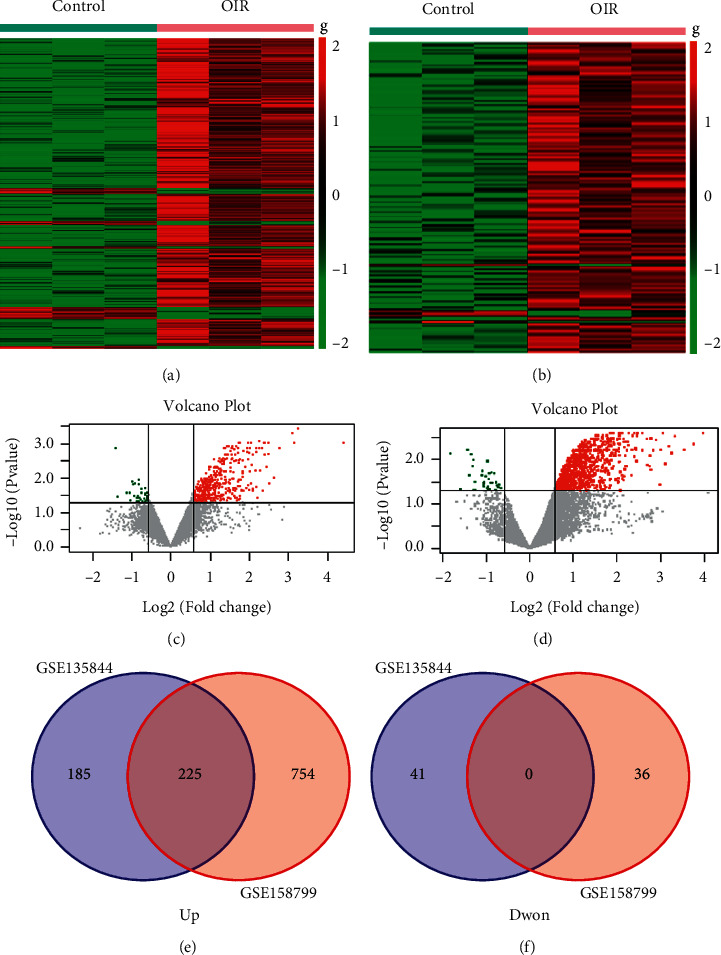
Heatmap of differentially expressed genes identified in (a) GSE158799 and (b) GSE135844. Legend on the top right indicates the log fold change of the genes; volcano plots of differentially expressed genes. (c) GSE158799 and (d) GSE135844. Data points in red represent upregulated, and data points in green represent downregulated genes. The differences are set as |log FC|>0.585. Venn diagram of common differentially expressed genes from the two datasets. (e) 0 DEG was downregulated in the two datasets, and (F) 225 DEGs were upregulated in the two datasets.

In the GSE135844 dataset, 451 DEGs were obtained, of which 410 were upregulated and the other 41 were downregulated in OIR; in the GSE158799 dataset, 1015 DEGs were found, of which 979 were upregulated and 36 were downregulated in OIR. Volcano maps and heatmaps were generated to visualize the clustering of the DEG expression, as shown in Figures 2(a)–2(d). There were 225 DEGs common to both datasets, all of which were elevated in OIR (Figures 2(e) and 2(f)); the intersection of the two sets of DEGs is shown in a Venn diagram.

### 3.2. GO and KEGG Analysis

**Figure 3:**
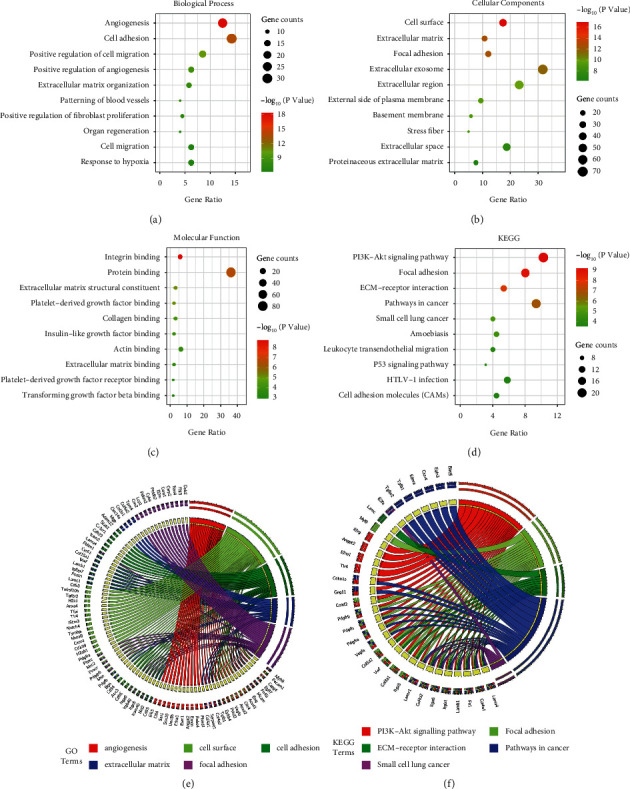
Gene Ontology (GO) and Kyoto Encyclopedia of Genes and Genomes (KEGG) enrichment analysis of differentially expressed genes (DEGs). The advanced bubble chart shows enrichment significance items of DEGs: (a) molecular function (MF), (b) biological processes (BP), (c) cell composition (CC), and (d) KEGG. The *x*-axis label represents the gene ratio, and the *y*-axis label represents GO or KEGG terms. The chord plot shows the distribution of DEGs in different (e) GO-enriched functions and (f) KEGG-enriched pathways. The size of the circle indicates the number of enriched genes.

Through GO analysis in the DAVID database, biological process (BP), cellular component (CC), and molecular function (MF) terms were analyzed, and the top ten terms are presented in a bubble chart. For BP, the results showed that the most enriched ones were related to angiogenesis, including angiogenesis (28 genes), positive regulation of angiogenesis (14 genes), and patterning of blood vessels (9 genes). Cell adhesion (32 genes), positive regulation of cell migration (19 genes), and extracellular matrix organization (13 genes) were also enriched. For CC, the enriched terms included cell surface (39 genes), extracellular matrix (24 genes), focal adhesion (27 genes), extracellular exosome (71 genes), and extracellular region (52 genes). For MF, the majority of enriched terms were related to binding, including protein binding (81 genes), calcium ion binding (19 genes), sequence-specific DNA binding (16 genes), and identical protein binding (16 genes). In the KEGG pathway analysis, the PI3K-Akt signaling pathway (23 genes) was the most enriched pathway. Focal adhesion (18 genes) was the second most enriched pathway in KEGG, which was consistent with the CC ontology results (Figures 3(d), 3(f)).

### 3.3. Gene Set Enrichment Analysis

**Figure 4:**
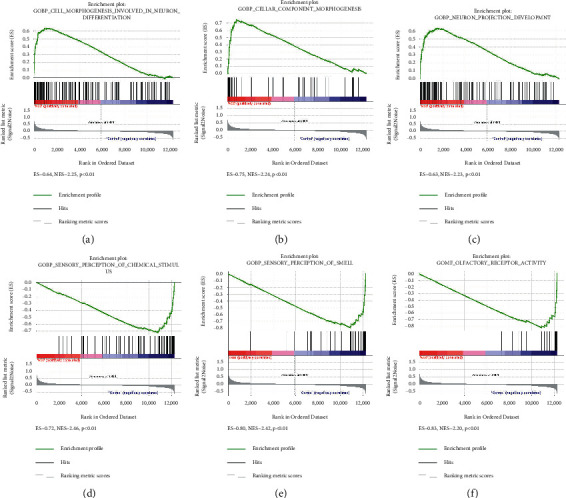
GSEA plots showing the most enriched gene sets of all detected genes in the ROP group and the control group in the GSE158799 dataset. The top 3 most significant upregulated enriched gene sets in the ROP group: (a) positive regulation of vasculature development, (b) establishment of endothelial barrier, and (c) blood vessel endothelial cell migration. The top 3 most significant upregulated enriched gene sets in the control group: (d) sensory perception of chemical stimulus, (e) sensory perception of smell, and (f) olfactory receptor activity.

The gene sets with significant differences between the OIR group and the control group were identified by GSEA. For the OIR group, the top 3 significantly enriched gene sets were “positive regulation of vasculature development,” “establishment of endothelial barrier,” and “blood vessel endothelial cell migration” (Figures 4(a)–4(c)). It was obvious that the most significant gene set was “angiogenesis,” consistent with the above GO analysis. Meanwhile, in the control group, “sensory perception of chemical stimulus,” “sensory perception of smell,” and “olfactory receptor activity” were the most significant gene sets (Figures 4(d)–4(f)).

### 3.4. PPI Network and Hub Gene Analysis

**Figure 5:**
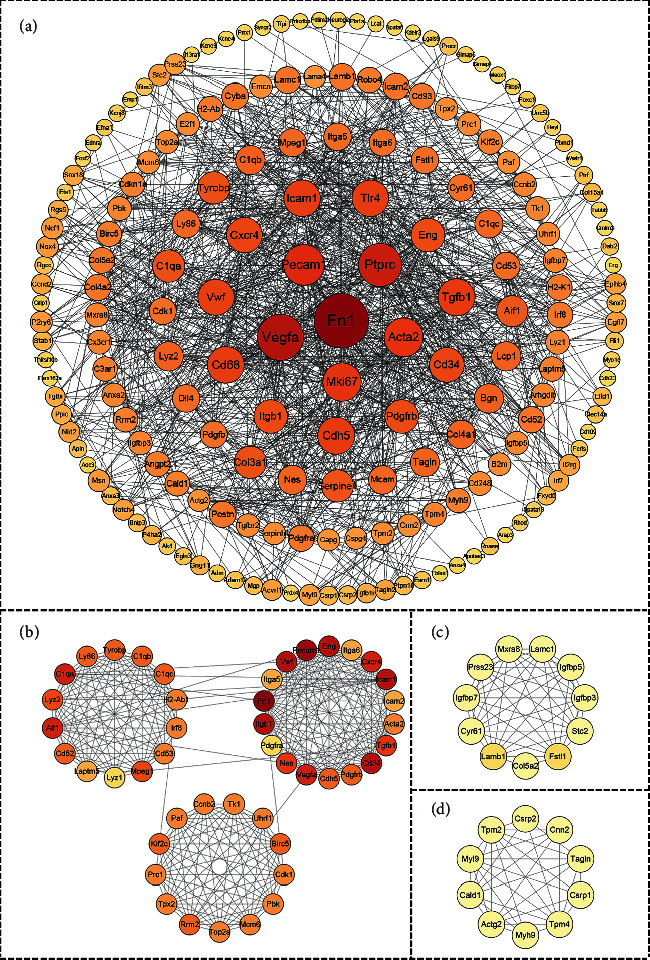
(a) PPI network of DEGs created by STRING. Circles represent genes, and lines represent PPIs. (b) The most significant module identified by MCODE (score = 13.773). (c) The second significant module identified by MCODE (score = 9.400). (d) The third significant module identified by MCODE (score = 7.556). DEG, differentially expressed gene; PPI, protein-protein interaction.

A PPI network was constructed by the online platform STRING. The PPI network contained 221 DEGs, with 221 nodes and 1424 edges (Figure 5(a)). The three most important modules were identified by MCODE (Figures 5(b)–5(d)). The key nodes in the network were discovered by CytoHubba. Finally, according to the degree, ten proteins, FN1, VEGFA, PTPRC, PECAM1, ACTA2, MKI67, TGFB1, TLR4, CD68, and CDH5, were selected as hub genes (Figure 5(a)).

### 3.5. Hub Genes Related to ROP

**Figure 6:**
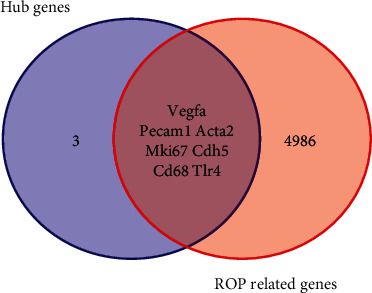
Identified 7 hub genes from the Comparative Toxicogenomics database.

The CTD indicates that there are 4993 genes known to be related to ROP. Interestingly, we found that 7 of these genes (VEGFA, PECAM1, ACTA2, MKI67, TLR4, CD68, and CDH5) overlapped with the 10 hub genes identified in our bioinformatics analysis (Figure 6). Finally, these 7 genes were selected as hub genes related to ROP.

### 3.6. Hub Genes Were Verified by RT-qPCR

**Figure 7:**
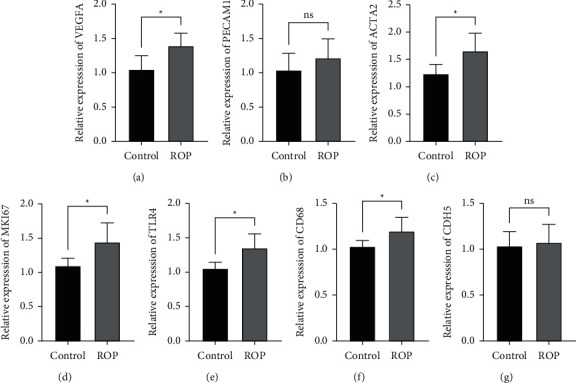
Differential expression of mRNAs in peripheral blood between ROP and control was validated by qRT-PCR. (a) VEGFA, (b) PECAM1, (c) ACTA2, (d) MKI67, (e) TLR4, (f) CD68, and (g) CDH5, ns (no significant difference, *P* > 0.05),  ^*∗*^*P* < 0.05,  ^*∗*^ ^*∗*^*P* < 0.01, and  ^*∗*^ ^*∗*^ ^*∗*^ ^*∗*^*P* < 0.0001.

The expression of these 7 genes in peripheral blood samples from 10 premature infants was measured by RT-qPCR. The expression of VEGFA, ACTA2, MKI67, TLR4, and CD68 in the ROP group was increased and showed a significant difference from that in the control group, while the other two genes (PECAM1 and CDH5) did not show a significant difference in expression (Figure 7). This finding indicated that VEGFA, ACTA2, MKI67, TLR4, and CD68 may play key roles in ROP.

### 3.7. TF-Gene and miRNA-Gene Networks

**Figure 8:**
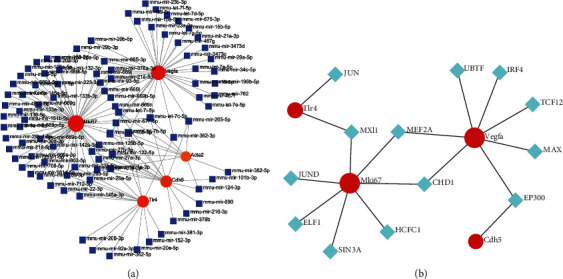
The networks of (a) miRNA-gene and (b) TF-gene. The red circle nodes are the genes, the blue square nodes are the miRNAs, and the green diamond nodes are the TFs.

To further explore the mechanism of ROP, we predicted miRNAs and TFs that may target and regulate core genes by NetworkAnalyst. The results identified 58 miRNAs regulating MKI67, 37 miRNAs regulating VEGFA, and 16 miRNAs regulating TLR4. Among them, mmu-let-7b-5p regulated the most core genes (5 genes) (Figure 8(a)). For TF genes, VEGFA andMKI67 were modulated by 7 TFs, as shown in Figure 8(b).

### 3.8. Drug-Gene Network

**Figure 9:**
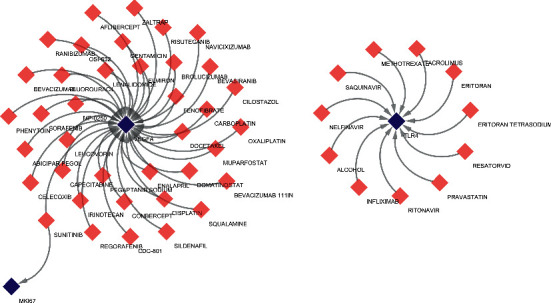
The drug-gene interaction network. The blue diamond nodes are the genes, and the red diamond nodes are the drugs.

Possible drugs or molecular compounds that can inhibit the upregulation of DEG expression in ROP were identified by searching CTD. Drugs or molecular compounds related to the above hub genes were identified. Among these drugs or molecular compounds, ranibizumab, which inhibits the expression of VEGFA, has been utilized in the treatment of ROP. Eritoran, a TLR4 antagonist, and sunitinib, a tyrosine kinase inhibitor that regulates the expression of MKI67, both showed high positive correlations with hub genes and may be considered potential targeted treatments for ROP (Figure 9).

## 4. Discussion

ROP is a disease of abnormal retinal vascular proliferation that is common in preterm infants. Great efforts have been made to explore the possible pathogenesis of ROP. However, uncontrolled neovascularization remains one of the major causes of blindness. A two-stage hypothesis for the pathogenesis of ROP is widely recognized [25, 26]. In stage 1, physiological vascular development is delayed, and vascular attenuation of the retina occurs due to hyperoxia and the absence of growth factors and nutrients. In stage 2, vascular proliferation occurs at the junction of the avascular and vascularized retina. Laser therapy, which has been widely applied for the treatment of ROP in the past few decades, remains controversial because of its side effects, which include high myopia, visual field loss, and retinal damage [27, 28]. In recent years, angiogenesis has been identified as one of the key mechanisms in the progression of ROP, and anti-VEGF therapy has been used in the clinical treatment of ROP, offering significant improvements in disease conditions and reducing the rate of laser therapy use [29, 30]. Unfortunately, anti-VEGF therapy also has drawbacks as a treatment for ROP. Intravitreal injection of anti-VEGF drugs into premature infants, who have a relatively small blood volume, results in a high drug concentration, which has raised concerns about its potential inhibition of retinal development of the retina and prevention of the systematic development of the organ system [31, 32]. Therefore, there is an urgent need to elucidate the biological mechanism to reduce and/or prevent adverse events in ROP. In our study, we attempted to comprehensively and thoroughly illustrate the genomic basis of ROP, identify hub genes, and uncover potential drugs.

A total of 225 common DEGs were identified between the OIR and normal groups based on the GSE158799 and GSE135844 datasets, and all were upregulated in OIR. The GO analysis showed that the DEGs were primarily enriched in angiogenesis, cell surface, cell adhesion, extracellular matrix, and focal adhesion. It is well known that angiogenesis is an essential component of proliferative retinopathy, and the results confirmed the significance of angiogenesis in ROP. In addition, the PI3K/Akt signaling pathway, focal adhesion, and ECM-receptor interaction were found to be significantly enriched via KEGG pathway analysis. Recent studies have also demonstrated that the PI3K/AKT pathway can directly and indirectly induce angiogenesis and may be intimately involved in ROP [33]. LY294002, an inhibitor of PI3K, prevented retinal neovascularization by downregulating the expression of VEGF, PI3K, and AKT both in vivo and in vitro [34]. We further performed GSEA and interestingly found that the main enriched gene terms were associated with positive regulation of vasculature development, blood vessel endothelial cell migration, and establishment of endothelial barrier, consistent with aspects related to angiogenesis highlighted in the GO analysis. Finally, a PPI network was constructed, and seven hub genes were identified: VEGFA, PECAM1, ACTA2, MK167, CDH5, CD68, and TLR4. RT-qPCR verification revealed that five of these hub genes (VEGFA, ACTA2, MK167, CD68, and TLR4) were highly expressed in premature infants with ROP.

VEGFA, a member of the PDGF/VEGF growth factor family, is required for both physiological and pathological vascularization and mainly induces the propagation and migration of vascular endothelial cells. Disruption of this gene leads to aberrant embryonic vascularization in mice [35]. Allelic mutation of the gene has been connected with microvascular complications of type 1 diabetes, which is a major cause of the progression of diabetic retinopathy [36]. Furthermore, numerous clinical studies have shown an increase in VEGF levels in the intraocular fluid of ROP patients, and intravitreal injection of anti-VEGF drugs could help decrease the level of VEGF [37–39]. These findings demonstrate the vital role of VEGF in the pathogenesis of ROP. The ACTA2 gene encodes smooth muscle actin, which is one of six different actins that participate in vasoconstriction and blood pressure homeostasis. Various vascular diseases are correlated with ACTA2 genetic variation. Ding et al. identified that exposure to high glucose results in epithelial-to-mesenchymal transformation (EMT) in retinal pigment epithelial (RPE) cells, which is one etiopathogenesis of DR, via coinstantaneous downregulation of E-cadherin and upregulation of alpha-smooth muscle actin, which is encoded by ACTA2 [40]. MK167 encodes a nuclear protein. It plays a role in cell proliferation and has been extensively used as a proliferation marker of human tumor cells for decades [41]. MK167 has also been used as a proliferation marker of endothelial cell proliferation in the human or mouse retina [42, 43]. CD68 is a member of the lysosome/endosomal cell-associated membrane glycoprotein family, with high expression in monocytes and macrophagocytes. Studies have shown that tumor infiltration by macrophages is closely related to angiogenesis; that is, angiogenesis will not occur in the absence of macrophages [44–46]. In addition, macrophages can secrete VEGF to facilitate blood vessel maturation and permeability [47, 48]. TLR4 belongs to the toll-like receptor (TLR) family and is mainly involved in pathogen recognition and innate immune activation. TLR4 is one of many inflammatory pathways in diabetic retinopathy. Devaraj et al. demonstrated that TLR4 actuates retinal inflammation in diabetic TLR4 knockout mice [49]. Compared with wild-type diabetic mice, TLR4 knockout mice exhibited improvements in diabetic retinopathy due to inhibition of the NF-kB signaling pathway and downstream inflammation, as well as downregulation of the expression of VEGF and GFAP [50]. A previous study also revealed that the loss of TLR4 can reduce inflammatory mediators and downregulate VEGF levels [51].

To further explore the pathogenesis of ROP, we constructed a miRNA-gene network that included 96 miRNAs and 5 hub genes. mmu-let-7b-5p regulates the most hub genes in the network, and further research regarding the relationship between mmu-let-7b-5p and ROP is clearly needed. In addition, we constructed a TF-miRNA network and discovered 13 TFs that may regulate the expression of hub genes. Four TFs (MXI1, MEF2A, EP300, and CHD1) simultaneously regulate 2 hub genes and were found to be closely related to VEGF expression, arterial vasculopathy, and endothelial cell proliferation [52–54]. These TFs may also become potential targets for studies of the mechanism of ROP.

To identify potential alternative pharmacological treatments for ROP, we performed a drug-gene interaction analysis. Our results found abundant gene-associated drugs, among which we mainly focused on VEGFA inhibitors, TLR4 antagonists, and sunitinib. Ranibizumab, a classical therapeutic agent for ROP, is a monoclonal antibody fragment against VEGFA, and clinical studies have shown that its treatment success rate for ROP is equivalent to that of laser therapy [28, 29]. The occurrence of systemic VEGF suppression and the long-term effects of ranibizumab treatment on vision are not yet known. Therefore, it is urgent to identify new potential drugs. TLR4 antagonists can efficiently block the inflammatory response in diseases such as corneal xenotransplantation and diabetic retinopathy [55]. Oxidative stress and inflammation are also recognized pathogeneses of ROP [56], which suggests that TLR4 antagonists may be promising drugs in ROP. Sunitinib, a tyrosine kinase inhibitor, is an antiangiogenic drug that has been used sparingly in ocular neovascular diseases such as von Hippel–Lindau disease and has shown a good therapeutic effect in case reports [57, 58]. Our study may offer significant new drugs for future therapeutic approaches to ROP.

In conclusion, we identified 5 key genes associated with ROP. To our knowledge, this is the first comprehensive study to integrate genes and DEGs and to identify and ultimately verify hub genes. Our findings offer novel insights into the role of potential biomarkers in ROP and identify candidate drugs for ROP therapy, which may have great clinical significance. However, the relatively small number of clinical samples tested is a limitation of this study, and further research is required to confirm our findings.

## Figures and Tables

**Table 1:** The primer sequence for PCR amplification.

Gene	Forward	Reverse
VEGFA	TGCAGATTATGCGGATCAAACC	TGCATTCACATTTGTTGTGCTGTAG
PECAM1	CTGATGCCGTGGAAAGCAGA	GGAGCAGGGCAGGTTCATAA
ACTA2	AGCCAAGCACTGTCAGGAAT	CACCATCACCCCCTGATGTC
MKI67	ACGCCTGGTTACTATCAAAAGG	CAGACCCATTTACTTGTGTTGGA
CDH5	ATTTGGGGATGACAGCCTTGG	TACTAACGGGTGGAACCTCAATAAC
CD68	CTACATGGCGGTGGAGTACAA	GAATGATGCTCGAGTTGCTGC
TLR4	AAGTTATTGTGGTGGTGTCTAG	GAGGTAGGTGTTTCTGCTAAG
GAPDH	TGCACCACCAACTGCTTAG	GATGCAGGGATGATGTTC

## Data Availability

The data used to support the findings of this study are available from the corresponding author upon request.
